# Supplementary material for: Positive narrativity enhances sense of agency toward a VR avatar
Source: Front Psychol. 2026 Mar 17;17:1775907. doi: 10.3389/fpsyg.2026.1775907 (PMC13036152; doi:10.3389/fpsyg.2026.1775907)
Supplement: Supplementary file 1 [file Table_1.docx]

Supplementary Material

**A Full Narrative Text for the Golem Avatar**

**A.1 Text presented to both groups**

The Golem is a giant figure made of materials such as clay that appears in folklore and mythology. For example, a legend from Prague, Czech Republic, a Golem was created from the mud of a large river to protect local residents from enemy attacks. Even in modern times, the Golem remains a well-known character that appears in roll-playing games (RPGs) and fantasy films, where it is often depicted as an artificial giant with a body made of rock or stone.
You will now listen to a narrative about the Golem that will be used as the avatar in today’s VR experience. The narrative will now begin.

**A.2 Text presented only to the Positive Group**

A certain village was repeatedly attacked by bandits from the surrounding mountains. To protect the village, the village sage created a golem. The golem obeyed the sage’s orders absolutely, accurately understood the defensive strategies for protecting the village from the bandits, and faithfully carried out attacks against approaching bandits. Because the golem could calmly assess situations, it was able to respond flexibly under any circumstances and take the most effective actions to protect the village. Its body was made of stone, which could withstand any kind of weapon. As a result, it defeated all the bandits who approached the village and kept the villagers safe. Through its unwavering loyalty to the sage, its calmness in all situation, and its immense power, the golem continued to protect the village from external threats. The villagers trusted the golem’s presence and were always grateful to it.

**A.3 Text presented only to the Negative Group**

A certain village was repeatedly attacked by bandits from the surrounding mountains. To protect the village, the village sage created a golem. The golem obeyed the sage’s orders absolutely, but it misunderstood the defensive strategies for protecting the village and sometimes attacked not only the bandits but also people who merely approached the village. Because the golem could not understand human emotions, it could not change its behavior in such situations and sometimes took actions that had the opposite effect of protecting the village. Its body was made of stone, which could withstand any kind of weapon. As a result, it not only defeated all the bandits who approached the village but also attacked villagers who tried to stop its mistaken actions. Although the golem was loyal to the sage, its inability to respond flexibly to situations, combined with its immense power, brought chaos to the village. The villagers came to fear the golem’s presence and gradually grew to hate it.

**B Questionnaire on Bodily Self-Consciousness (SoO and SoA)**

**Table1. Questionnaire on Bodily Self-Consciousness**

| Items | Questionnaire items |
| --- | --- |
| Sense of Ownership | 1. I felt as if I were looking at my own body. |
|  | 1. I felt as if the avatar (the golem)’s body were my own body. |
| Sense of Ownership (Dummy Items) | 1. I felt as if my own body had changed into the avatar (the golem). |
|  | 1. I felt as if I had more than one body. |
| Sense of Agency | 1. I felt as if the avatar (the golem)’s body moved just as I intended, following my will. |
|  | 1. I felt as if I were controlling the movements of the avatar (the golem)’s body. |
| Sense of Agency (Dummy Items) | 1. I felt as if the avatar (the golem)’s body was controlling my will. |
|  | 1. I felt as if the avatar (the golem)’s body was controlling my movements. |

**C Item–Item Correlations for SoO and SoA**

**Table2. Item–Item Correlations**

| Items | N | Spearman’s ρ | 95% Cl | p-value |
| --- | --- | --- | --- | --- |
| Sense of Ownership | 32 | 0.65 | [0.30,0.87] | <.001 |
| Sense of Agency | 32 | 0.35 | [-0.04,0.67] | .048 |
